# A systematic review and meta-analysis protocol on stunting and its determinants among school-age children (6-14years) in Ethiopia

**DOI:** 10.1371/journal.pone.0248390

**Published:** 2021-03-18

**Authors:** Setognal Birara Aychiluhm, Abay woday Tadesse, Kusse Urmale Mare, Dessie Abebaw, Netsanet Worku

**Affiliations:** 1 College of Medicine and Health Sciences, Department of Public Health, Samara University, Samara, Ethiopia; 2 College of Medicine and Health Sciences, Department of Nursing, Samara University, Samara, Ethiopia; 3 College of Medicine and Health Sciences, Institute of Public Health, Department of Epidemiology & Biostatistics, University of Gondar, Gondar, Ethiopia; 4 College of Medicine and Health Sciences, Institute of Public Health, Department of Nutrition, University of Gondar, Gondar, Ethiopia; Mansoura University, EGYPT

## Abstract

**Background:**

In Ethiopia, stunting is a common public health problem among school-age children. Even though several studies were conducted in different parts of the country, the national pooled prevalence of stunting and its determinants not estimated. Therefore, this study intends to determine the pooled prevalence and determinants of stunting among school-age children in Ethiopia.

**Methods:**

This review protocol is registered at PROSPERO with Registration number: CRD42020160625. Online databases (Medline, PubMed, Scopus, and Science direct), Google, Google Scholar, and other grey literature will be used to search articles until June 2020. The quality assessment will be performed using the Joanna Briggs Institute checklist. The analysis will be organized and presented according to the Preferred Reporting Items for Systematic Reviews and Meta-Analysis. The presence of heterogeneity among studies will be examined using a chi-squared test on Cochran’s Q statistic with a 5% level of statistical significance, subgroup analyses, and meta-regression will be performed to investigate sources of heterogeneity. To identify influential studies, sensitivity analysis will be conducted. Presence publication bias will be examined by observing funnel plots. The presence of a statistical association will be declared at a p-value <0.05 with the 95% CI.

**Discussion:**

Stunting is a major public health problem in Ethiopia, which affects the health of children. So, designing and implementing different nutritional strategies and promoting healthcare services is extremely mandatory to overcome stunting problems in the country. To understand this, estimating the prevalence of stunting at the national level and determining the pertinent common determinants using high-level evidence is fairly imperative. Therefore, this study will offer a summarizing finding.

## Background

Child growth is an important indicator of nutritional status and health in populations. Stunting is the commonly used indicator of child growth among the three anthropometric indices [1]. According to the World Health Organization (WHO) growth reference standard, stunting is defined as height for age Z (HAZ) scores below -2 Z-score value [2].

Globally, stunting is one of the most serious and challenging public health problems in the world. It affects 165 million under-five children, of these 90% of them live in Asia and Africa [3], making it the main source of concern in developing countries more than underweight or wasting.

The burden of childhood stunting reduced from 39.7% in 1990 to 26.7% in 2010 [4]. Despite this improvement, approximately one-third of all children living in South Asia and sub-Saharan Africa were stunted in the year 2017 [4, 5].

In 2010, 38% of children were stunted in Africa [6], and 45% of school-aged children in Eastern Africa were stunted [4]. The trend has not shown a significant decrease, so that, still stunting is a major public health problem in developing countries by increasing the risk of child mortality [7–9].

Stunting results serious complications in children like reduced cognitive performance and development, poorer school attendance, low intellectual and physical abilities in adulthood, and reduced adult earning capacity [8, 10–14].

By 2030, Ethiopia planned to reach the zero-level under-nutrition status, and the government has been applying some strategies like the 2004 National Strategy for IYCF practices, the 2005/2006 National Nutrition Strategy, and the 2008 National Nutrition Program to reduce the burden of under-nutrition [15–17].

Despite this, the prevalence of stunting among school-age children ranges from 9.8% to 48.1%, which indicated that stunting is a public health problem in the country [11, 18].

In Ethiopia, there have been individual and inconclusive studies conducted to date to generate information regarding the prevalence and its determinants of stunting among school-age children.

There has been regional variation in prevalence in those individual studies, and determinants that contribute to these discrepancies are not documented at the national level. Therefore, this systematic review study attempts to estimate the pooled estimate and its determinants of stunting among school-age children at the national level.

### Research questions

1. What is the pooled prevalence of stunting among school-age children in Ethiopia?
2. What are the major determinants of stunting among school-age children in Ethiopia?

### Objectives

1. To determine the pooled prevalence of stunting among school-age children in Ethiopia
2. To identify determinants of stunting among school-age children in Ethiopia.

## Methods

### Registration and reporting of the review findings

The protocol for this review is registered at PROSPERO with registration number: CRD42020160625. We will use the Preferred Reporting Items for Systematic review and Meta-analyses (PRISMA-P 2009) [19], and the Meta-analysis of Observational Studies in Epidemiology [20] and (PRISMA-P 2015) [21] (*Additional file 1 in* S1 File) statements to report the findings.

### Study design

Systematic review and meta-analysis will be employed to determine the pooled prevalence of stunting and its determinants among school-age children in Ethiopia.

### Eligibility criteria

#### Inclusion criteria

- All observational studies, including cross-sectional, analytical cross-sectional, case-control, and cohort studies.
- All articles published only in the English language.
- All articles which were done in Ethiopia and reporting the prevalence and associated factors of stunting will be included without time restriction.

#### Exclusion criteria

- Articles without full text and data that are difficult to extract
- Children whose age group are not well defined
- studies published in languages other than English
- studies other than observational studies like Case reports, conference reports, national survey reports, and expert opinions

#### PECO search guide

*Population*. School-age children (6–14 years old).

*Exposure*. Predictors or determinants of stunting. The determinants are characteristics or exposures that increase the likelihood of stunting among school-age children. Those factors include residence site, educational status of mothers or caregivers, sex of the child, age of the child, and household food security status.

*Comparison*. The reported reference group for each determinant or associated factors in each study (e.g. stunting in children residing in an urban area versus rural area, children from food secured versus food in secured households).

*Outcome*. We will include studies that assess the prevalence of stunting and its determinants among school-age children in Ethiopia.

#### Searching strategy

Preferred Reporting Items for Systematic Reviews and Meta-Analysis will be used for the preparation and presentation of this meta-analysis. Online databases (Medline, PubMed, Scopus, and Science direct), Google, Google Scholar, and other grey literature will be used to search articles until June 2020. The searching strategy for PubMed online database is added as a supplementary file with this protocol (*Additional file 2 in* S1 File). Snowballing will be used to screen the references of identified articles for possibly relevant studies. Studies identified by our database searching strategy will be retrieved and managed using Endnote X8 software [22].

Medical Subject Heading (Mesh), keywords, and free text search terms will be used. As the search terms, we will include alternative terms for stunting and will combine them using Boolean operators

*Search terms*. Search (*((“Stunting” OR “Stunt” OR “*Growth and Development” OR “Growth” OR “Body Size” OR “Body Height” AND *“school-age children” OR “6–14 years old children”) AND (Prevalence OR burden OR magnitude OR incidence) AND (predictors OR “risk factors” OR determinants OR associated factors OR causes”) AND (Ethiopia OR Etiopia OR Ethio))*)

#### Outcome measurement

According to the WHO growth reference standard, Stunting is defined as height for age Z (HAZ) scores below -2 Z-score value [2].

#### Selection of studies

Two authors (SB and AW) will review the studies, based on inclusion and exclusion criteria. At the first stage, relevant articles will be considered based on their title.

Abstracts of these selected titles will be incorporated at the second stage. In the third stage, the full-text screening will be conducted. In case, if articles are not open access, we will contact the corresponding author at least three times. If the authors are not willing to provide the full text, that specific article will be excluded from the study.

Studies that are approved by both authors in the review processes will be included. Any differences in ideas will be resolved through discussion until an agreement is reached. A third reviewer (NW) may be consulted if disagreement happens between the two reviewers. Lastly, we will organize a final list of articles for data extraction

#### Data extraction and management

After all eligible articles are identified, two independent reviewers (SB and DA) will extract the relevant data using an organized format on Microsoft Excel Spreadsheet.

Discrepancies between data extractors will be discussed to reach a consensus. If a consensus cannot be reached, the authors will consult a third reviewer (NW). For each included article, we will record the first author’s last name, year of publication, the setting where the study was conducted, study design, study period, sample size, the response rate, the population, outcome definition, comparison groups, and pertinent associated factors, and the effect estimate.

For prevalence studies, prevalence, the logarithm of prevalence, and SE of the logarithm of prevalence will be calculated. Similarly, for determinants, OR, logarithms of OR, and SE of the logarithms of OR will be calculated. For any difficulties that might be encountered during data extraction, the corresponding author(s) will be communicated.

#### Quality assessments

The quality assessment will be conducted by five authors (SB, AW, NW, KU, and DA) using the checklist of the JBI appraisal tool for cross-sectional, cohort, and case-control studies (Additional file 3 in S1 File). This tool will include different questions based on the study designs. For studies with cohort, case-control, and analytical cross-sectional study designs, question items 10, 8, and 11 will be employed, respectively. For studies with simple cross-sectional studies, a tool with nine-question items will be used. The tools have ‘Yes’ and ‘No’ types of questions and scores will be given 1 for ‘Yes’ and 0 for ‘No’ responses. Scores will be summed and converted into a percentage.

Only studies that scored ≥50% will be considered for both systematic review and meta-analysis of prevalence. For any scoring disagreements, which might happen between the abstractors, the sources of discrepancy will be investigated through revision. If disagreements persist, after the detailed review, the average scores of the reviewers will be calculated. In the same way, for determinants, each determinant with the outcome variable will be critically evaluated. A similar cut-off point that we will be using for prevalence studies will be applied to determinants of stunting.

#### Data synthesis and analysis

The extracted data will be imported into STATA version 16 (Stata Corp LLC, Texas, USA) software. A Narrative description of the study population will be performed. Tables and figures will be used to summarize the selected studies and results.

The pooled prevalence of stunting among school-age children in Ethiopia will be demonstrated using the random effect model [23]. The Freeman Tuckey variant of the arcsine square root transformation of proportions will be used to avoid variance variability when controlling proportions close to one [24]. We will assess heterogeneity by using the chi-squared test on Cochran’s Q statistic with a 5% level of statistical significance [25] and I^2^ statistic test [26], assuming that I^2^ value of 25%, 50%, and 75% is representative of low, moderate, and high heterogeneity, respectively [26]. If the heterogeneity is significant (I^2^ > 75%) and p-value < 0.05 will be declared as the presence of heterogeneity. Thus, subgroup analyses and meta-regression will be performed to investigate sources of heterogeneity.

To identify influential studies, sensitivity analysis will be conducted [25]. Publication bias will be examined by the visual inspection of funnel plots [27] and Egger’s test [28]. A p-value < 0.10 will be considered indicative of statistically significant publication bias, if evidence of publication bias present, thus the trim-and-fill (Duval and Tweedie’s) method will be performed [29].

The existence of an association between the determinants and stunting will be estimated based on the effect size. Then, the statistical significance level will be declared at a p-value of less than 0.05.

## Discussion

Stunting among children has lifelong implications, understanding the stunting status of children will have far-reaching implications. Its outcomes not only cover the whole life but also transferred from one generation to another [30].

In Ethiopia, the prevalence of stunting among school-age children ranges from 9.8% [11] to 48.1% [18]. This implies it is a major public health problem in the country, which affects the health of children. So, designing and implementing different nutritional strategies and promoting healthcare services is extremely mandatory to fire stunting problems in the country.

To understand this, estimating the prevalence of stunting at the national level and determining the pertinent common determinants using high-level evidence is imperative. Therefore, this study will offer a summarizing finding.

As a limitation, heterogeneity is predictable in this meta-analysis as we will consider a variety of study designs and from different geographic areas of the country. The other limitation is that the search strategy will be restricted articles published only in the English language but there might be articles that published using another language. This systematic review considers only observational studies and excludes randomized clinical trials and quasi-experimental studies that are the gold standard.

## Supporting information

S1 File (DOCX)
